# Carbon ion radiotherapy for basal cell adenocarcinoma of the head and neck: preliminary report of six cases and review of the literature

**DOI:** 10.1186/1748-717X-5-89

**Published:** 2010-10-04

**Authors:** Keiichi Jingu, Azusa Hasegawa, Jun-Etsu Mizo, Hiroki Bessho, Takamichi Morikawa, Hiroshi Tsuji, Hirohiko Tsujii, Tadashi Kamada

**Affiliations:** 1 Research Center for Charged Particle Therapy, National Institute of Radiological Sciences (NIRS), Chiba, Japan

## Abstract

**Background:**

Basal cell adenocarcinoma accounts for approximately 1.6% of all salivary gland neoplasms. In this report, we describe our experiences of treatment for BCAC with carbon ion radiotherapy in our institution.

**Methods:**

Case records of 6 patients with diagnosis of basal cell adenocarcinoma of the head and neck, who were treated by carbon ion radiotherapy with 64.0 GyE/16 fractions in our institution, were retrospectively reviewed.

**Results:**

In a mean follow-up period of 32.1 months (14.0-51.3 months), overall survival and local control rates of 100% were achieved. Only one grade 4 (CTCAE v3.0) late complication occurred. There was no other grade 3 or higher toxicity.

**Conclusions:**

Carbon ion radiotherapy should be considered as an appropriate curative approach for treatment of basal cell adenocarcinoma in certain cases, particularly in cases of unresectable disease and postoperative gross residual or recurrent disease.

## Background

Basal cell adenocarcinoma (BCAC) was first recognized in 1978 and accounts for approximately 1.6% of all salivary gland neoplasms [1]. BCAC typically arises in adults older than 60 years of age and has no gender predominance [2]. The vast majority of BCACs occur in the parotid gland (about 90%) [3-5], followed by the submandibular gland and minor salivary glands [6]. The 2005 WHO classification categorizes BCAC as a low-grade tumor with a favorable prognosis [7]. The standard treatment has been wide local excision with or without postoperative radiotherapy. However, local recurrence has frequently been reported.

Carbon ion radiation therapy (C-ion RT) was initiated at the National Institute of Radiological Sciences (NIRS) in 1994 [8]. For malignant tumors of the head and neck, a phase II clinical trial with C-ion RT was started in April 1997. So far, we have treated more than 350 patients with a large histological variety of malignant tumors of the head and neck including mainly mucosal malignant melanoma and adenoid cystic carcinoma. Of those patients, 6 patients with BCAC of the head and neck were enrolled. In this report, we describe the 6 patients with BCAC and the efficacy and complications of C-ion RT.

## Methods

### Case Presentation

The 6 patients' characteristics are shown in Table 1. Mean age was 58 years (range: 37-81 years). None of the patients had metastasis in distant organs. The primary sites were parotid gland in 4 patients, base of the tongue in 1 patient and ethmoid sinus in 1 patient. The stages for all patients were defined according to Unio Internationalis Contra Cancrum (UICC) 2002. Histology of all patients was reconfirmed by a pathologist in our institution before C-ion RT.

**Table 1:** Patients' Characteristics

Patient	Age	Gender	Primary Site	Stage (UICC§ 2002)	Tumor Response (RECIST*)	Grade 3 or more Toxicities (CTCAE† v3.0)	Observation Period (months)
1	43	M	base of tongue	cT4aN0M0	PR	none	25.9

2	70	M	ethmoid sinus	cT4aN0M0	PR	Grade 4 retinopathy	20.9

3	62	F	parotid grand	postoperative recurrence (pT3N0M0, R0)	CR	none	14.0

4	37	F	parotid grand	cT3N1M0	PR	none	49.6

5	81	M	parotid grand	cT4aN0M0	SD	none	51.3

6	55	M	parotid grand	postoperative residual (pT4aN0M0, R2)	CR	none	31.3

Abbreviation, §Unio Internationalis Contra Cancrum; *Response Evaluation Criteria in Solid Tumors; †Common Terminology Criteria for Adverse Events

### Clinical Histories

#### Patient 1

A 43-year-old Japanese male developed a sore throat over a period of 3 months. A tumor at the base of the tongue was detected by endoscopy. The pathological diagnosis was BCAC by biopsy. CT revealed that the clinical stage was T4aN0M0 (stage IVA). The diameter of the primary tumor was 29 mm. At first, one cycle of chemotherapy, including cisplatin, 5-FU and docetaxel, was performed in the previous hospital; however, the tumor did not show shrinkage. He therefore came to our institution for C-ion RT.

#### Patient 2

A 70-year-old Japanese male had nasal bleeding for one week. A tumor in the right ethmoid sinus was detected by endoscopy and CT in the previous hospital. Biopsy was performed in the previous hospital, and the diagnosis was BCAC (MIB-1 index, 50-80%) in the right ethmoid sinus with intracranial invasion. The diameter of the primary tumor was 50 mm and there was no lymphadenopathy (cT4aN0M0, stage IVA). There was no indication for surgery. He came to our institution for C-ion RT. The patient had bilateral retinal detachments as a past history.

#### Patient 3

A 62-year-old Japanese female had undergone right total parotidectomy in the previous hospital (pT3N0M0, stage III, R0). The pathological diagnosis was BCAC. Thereafter, follow-up was performed every 3 months. Eight years after parotidectomy, a tumor of 54 mm in diameter was detected under the right temporal skin by MRI, and BCAC recurrence was confirmed by biopsy. No lymphadenopathy was detected. There was no indication for surgery. She came to our institution for C-ion RT.

#### Patient 4

A 37-year-old Japanese female developed fullness in the right ear and right buccal swelling over a period of 3 months. She underwent fine needle biopsy and was diagnosed as cytologic class III in the previous hospital. Total parotidectomy +/- postoperative radiotherapy was planned. CT revealed that the clinical stage was T3N1M0 (stage III). The diameter of the primary tumor was 54 mm and the diameter of the right upper cervical lymph node was 18 mm. However, she declined surgery and requested C-ion RT. We required the previous hospital to perform biopsy for confirming the histology. Thereafter, her tumor was diagnosed as BCAC (MIB-1 index, 10%).

#### Patient 5

An 81-year-old Japanese male developed left buccal swelling over a period of one and half years. A benign tumor was suspected by CT, but the histological diagnosis was BCAC by biopsy. The clinical stage was T4aN0M0 (stage IVA). The diameter of the primary tumor was 52 mm and there was no lymphadenopathy. If curative surgery was performed, facial nerve palsy could not be avoided. For this reason, he declined curative surgery and selected C-ion RT.

#### Patient 6

A 55-year-old Japanese male had right buccal swelling. A benign tumor was suspected and observation was performed. Four years later, a gastric malignant tumor was found by medical examination. Right partial parotidectomy was performed simultaneously with total gastric resection. The histological diagnosis of the parotid tumor was BCAC with suspected residual macroscopic tumor (pT4aN0M0, stage IVA, R2). For gastric cancer, chemotherapy including TS-1 was performed for 6 months after surgery. However, a gross tumor of 19 mm in diameter in his right parotid gland remained. He selected C-ion RT.

### Treatment

All of the patients were not indicated for curative surgery or declined surgery, and C-ion RT was performed as follows.

### Carbon Ion Radiotherapy

Doses of carbon ions were expressed in photon equivalent doses (GyE), which were defined as the physical doses multiplied by the RBE of the carbon ions. The biological flatness of the SOBP was normalized by the survival fraction of human salivary gland tumor cells at the distal region of the SOBP, where the RBE of carbon ions was assumed to be 3.0 [9].

The patients were positioned in customized cradles (Moldcare; Alcare, Tokyo, Japan) and immobilized with a low-temperature thermoplastic shell (Shellfitter; Kuraray, Osaka, Japan). A set of 2.5-mm-thick computed tomography (CT) images was taken for treatment planning with the immobilization devices. CT imaging alone is inadequate for detection of extension of the tumor. Therefore, MRI was routinely used for identification of the tumor, after fusing it with the planning CT. Determination of gross target volume (GTV) was based on contrast-enhanced MRI. The clinical target volume (CTV) had minimum margins of 5.0 mm added around the GTV. The planning target volume (PTV) included margins of 3.0-5.0 mm around the CTV, and this could be modified manually. The PTV and OAR (e.g., eyeball wall, optic nerve, optic chiasma and brain stem) were outlined on the planning CT images to permit dose-volume histogram (DVH) analysis. Three-dimensional treatment planning was performed using HIPLAN software (National Institute of Radiological Sciences, Chiba, Japan) [10]. The PTV was ensured with at least 95% of the prescription dose.

Irradiation was carried out once per day for 4 days per week (Tuesday-Friday) with carbon ion beams. The prescribed dose to the center of the CTV was 64.0 GyE/16 fractions over 4 weeks at 4.0 GyE/fraction per day in all of the 6 patients. Thereafter, no other treatments were performed for any patients.

### Follow-up

The patients were followed up by CT or MRI every 1 or 2 months for the first 6 months after C-ion RT and thereafter every 3 to 6 months. The overall survival and local control rates were calculated from the first day of C-ion RT. Toxicities were classified according to Common Terminology Criteria for Adverse Events (CTCAE) v3.0.

## Results

**Figure 1:**
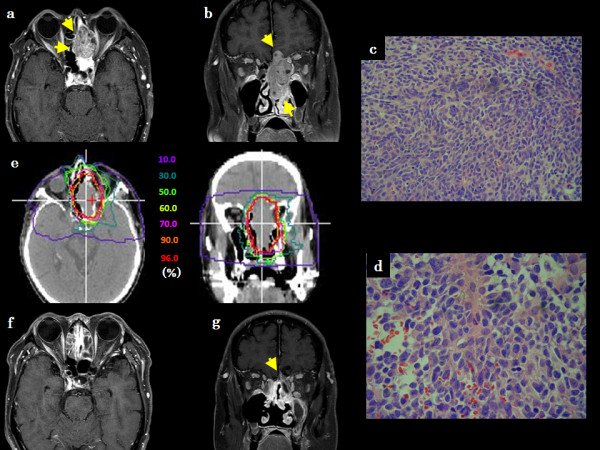
**Patient 2, a 70-year-old Japanese male with BCAC in the ethmoid sinus**. (a) Axial contrast-enhanced T1-weighted MR image before C-ion RT, (b) Coronal contrast-enhanced T1-weighted MR image before C-ion RT, (c) Histological findings of HE staining at low-magnification, (d) Histological findings of HE staining at high-magnification, (e) Dose-distribution of C-ion RT in axial and coronal CT images, (f) Axial contrast-enhanced T1-weighted MR image 1 year after C-ion RT, (g) Coronal contrast-enhanced T1-weighted MR image 1 year after C-ion RT.

**Figure 2:**
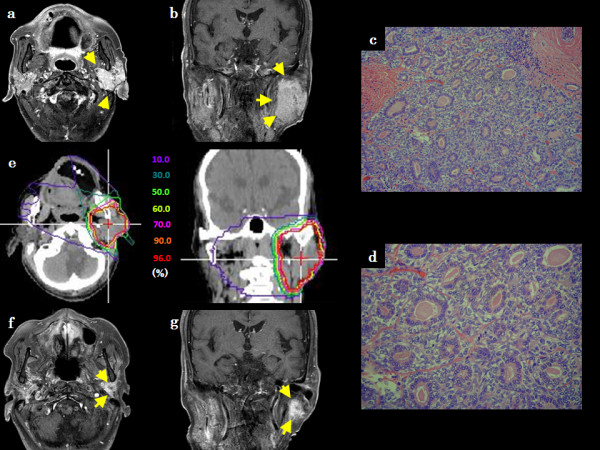
**Patient 5, an 81-year-old Japanese male with BCAC in the left parotid gland**. (a) Axial contrast-enhanced T1-weighted MR image before C-ion RT, (b) Coronal contrast-enhanced T1-weighted MR image before C-ion RT, (c) Histological findings of HE staining at low-magnification, (d) Histological findings of HE staining at high-magnification, (e) Dose-distribution of C-ion RT in axial and coronal CT images, (f) Axial contrast-enhanced T1-weighted MR image 3 years after C-ion RT, (g) Coronal contrast-enhanced T1-weighted MR image 3 years after C-ion RT.

All of the patients underwent C-ion RT without an interval, and all of the patients were alive at the last observation date. No patient was lost to follow-up. The mean observation period was 32.1 months (range: 14.0-51.3 months). There were no local or regional recurrences or metastasis in distant organs. Tumor response rate according to Response Evaluation Criteria in Solid Tumors (RECIST) was 66.7%, including 1 CR, 3 PR and 2 SD, at 6 months after completion of C-ion RT. MR images of 2 representative patients before and after C-ion RT and dose-distributions of C-ion RT are shown in Figures 1 and 2, respectively.

One patient who had a tumor in the left ethmoid sinus had grade 4 left retinopathy (light perception) about 12 months after completion of C-ion RT. Three of the 4 patients who had a tumor in the parotid gland did not show facial nerve palsy; however one patient showed slight facial nerve palsy 6 months after C-ion RT. There was no other grade 3 or higher toxicity in the 6 patients.

## Discussion

Since BCAC seldom metastasizes to cervical lymph nodes, routine neck dissection is not recommended. The mortality rate for this tumor is also low, although reported local recurrence rates are high. In a review, local recurrence was observed in 37% (17/46) of patients with follow-up between 6 months and 2 years [2]. In another review, local recurrence was observed in 44.4% (8/18) of patients with follow-up between 2 years and 14.3 years [11]. From the above experiences, it would appear that the first treatment of choice for BCAC is wide local excision with frozen-section control of the resection margins. However, sufficient resection margins often cannot to be obtained due to the need for preservation of critical organs (e.g., the facial nerve in parotid tumors). Therefore, postoperative radiotherapy has been proposed for lesions with a high risk of vascular and neural invasion and for lesions that are diffusely infiltrative, especially in patients with close resection margins [12]. Even with wide local excision and postoperative radiotherapy, local recurrence has been reported in about 30-50% of patients (Table 2) [2,11,13]. To our knowledge, this is the first report of BCAC treated with radiation alone. Although observation period of the present cases was not enough, C-ion RT achieved good local control among past reports. A possible explanation for the success we have seen with C-ion RT of BCAC concerns the differences in biological interactions of carbon ion radiation and photon radiation in tissue. Compared to photon radiation, high linear energy transfer (LET) radiation is characterized by less variation of sensitivity through the cell cycle [14], by less or no repair of sublethal or potentially lethal cell damage, which is a problem in controlling repair-proficient photon-resistant tumors, and by a reduced oxygen enhancement factor (OER) in the case of hypoxic and poorly-reoxygenating tumors. An indolent tumor such as BCAC with consequent ability to repair potentially lethal damage from low LET radiation might have an increased responsiveness to C-ion RT. High LET radiation, including C-ion RT, could be a favorable curative treatment for BCAC. More long-term observation is required.

**Table 2:** Literature Review of Treatment Results for Basal Cell Adenocarcinoma

Author	n	Observation Period (mean)	Treatment	Local Recurrence
Muller et al. [2]	7	5-192 months (54 months)	Surgery +/- X-ray	2/7

Parashar et al. [11]	18	2-14.3 years (5.1 years)	Surgery +/- X-ray	8/18

Nagao et al. [13]	10	1-18 years (6.5 years)	Surgery +/- X-ray	5/10

current series	6	14.0-51.3 months (32.1 months)	Carbon ion radiotherapy	0/6

With regard to toxicities, severe unilateral retinopathy occurred in one patient (patient 2) even with excellent dose-distribution of C-ion RT since the critical organ was next to the tumor. We have already revealed the dose constraints of optic nerves for C-ion RT [15]. Severe retinopathy was considered to be unavoidable in that patient. Grade 3 or more toxicity was observed in only that patient. Brown et al. reported that severe facial nerve palsy occurred in 26% of 66 patients who underwent surgery even with facial nerve graft for a parotid neoplasm and postoperative radiotherapy, [16]. On the other hand, Buchholz et al. reported that facial nerve palsy occurred in one of 6 patients with recurrent pleomorphic adenoma treated by fast neutron radiotherapy, which is also high LET radiation [17]. Duthoy et al. reported that decrease of vision occurred in 5 of 39 patients with sinonasal carcinoma treated with postoperative intensity-modulated radiation therapy [18]. Compared with those treatment methods, C-ion RT is considered to be acceptable. However, the average time of progression to eventual radiation-induced visual loss was 25.6 months (range, 10-41 months) after C-ion RT in our previous investigation [15]. Although facial nerves are considered to be stronger than optic nerves for C-ion RT since peripheral nerves are known to have more radio-resistance than central nerves [19], more facial nerve palsy in patients with a tumor in the parotid gland may occur in the long term. The acceptable dose of C-ion RT for facial nerves is currently under investigation.

## Conclusions

We reported preliminary but excellent efficacy of C-ion RT for BCAC, which is very rare head and neck malignant tumor, in 6 patients. Our results showing acceptable toxicities and appreciable efficacy suggest that C-ion RT could be one of the curative primary treatments of BCAC.

## Consent

Written consent for publication was obtained from all of the patients before C-ion RT in our institution.

## Competing interests

The authors declare that they have no competing interests.

## Authors' contributions

KJ and AH conceived the idea, did the literature search and prepared the manuscript. KJ, AH, JM, HB, TM and HT performed treatment and follow-up and acquisition of data. TK and HT provided critical review of the manuscript and research guidance. All authors read and approved the final manuscript.
